# Changes in Cytokine Expression after Electroacupuncture in Neuropathic Rats

**DOI:** 10.1155/2012/792765

**Published:** 2012-02-08

**Authors:** Myeoung Hoon Cha, Taick Sang Nam, Yongho Kwak, Hyejung Lee, Bae Hwan Lee

**Affiliations:** ^1^Department of Physiology and Brain Korea 21 Project for Medical Science, Yonsei University College of Medicine, P.O. Box 8044, Seoul 120-752, Republic of Korea; ^2^Acupuncture and Meridian Science Research Center, Kyung Hee University, Seoul 130-701, Republic of Korea

## Abstract

The production of proinflammatory cytokines including interleukin-1 (IL-1), interleukin-6 (IL-6), and tumor necrosis factor-**α** (TNF-**α**) plays a key role in chronic pain such as neuropathic pain. We investigated changes in cytokine expression in injured peripheral nerves and dorsal root ganglia (DRG) following electroacupuncture (EA) treatment. Neuropathic pain was induced by peripheral nerve injury to the left hind limb of Sprague-Dawley rats under pentobarbital anesthesia. Two weeks later, the nerve-injured rats were treated by EA for 10 minutes. The expression levels of IL-1**β**, IL-6, and TNF-**α** in peripheral nerves and DRG of neuropathic rats were significantly increased in nerve-injured rats. However, after EA, the cytokine expression levels were noticeably decreased in peripheral nerves and DRG. These results suggest that EA stimulation can reduce the levels of proinflamtory cytokines elevated after nerve injury.

## 1. Introduction

Acupuncture has been widely used in traditional East Asian medicine for the clinical treatment of chronic pain and various diseases such as rheumatoid arthritis and inflammatory bowel syndrome [1], but the mechanism of acupuncture-induced analgesia remains unclear. Recent studies have documented the analgesic effects of acupuncture and electroacupuncture (EA) stimulation using behavioral and molecular biological methods in rats [2–6].

Holguin et al. [7] observed that the expression of proinflammatory cytokines was increased by the release of nitric oxide. In addition, spinal nitric oxide improves pain facilitation through glial activation [8] and the release of interleukin-1*β* (IL-1*β*), interleukin-6 (IL-6), and tumor necrosis factor-*α* (TNF-*α*) [9]. Recently, interest in neuroinflammation and neuroimmune activation caused by neuropathic pain has grown rapidly. Inflammation and immune responses are caused by neurological disorders such as peripheral nerve injuries that are often associated with persistent pain [10]. Painful nerve injury results in rapid and sustained upregulation of IL-1*β*, IL-6, and TNF-*α* in the damaged nerve itself and in macrophages in the dorsal root ganglion (DRG) [11]. IL-1, IL-6, and TNF-*α* have been known to play an important role in the inflammatory response to pain [12–14]. In particular, Lee et al. [14] suggest that IL-1*β* and TNF-*α* function in the initiation of persistent neuropathic pain, while IL-6 is important for maintenance. However, the relationship between acupuncture analgesia and expression in inflammatory cytokines is unclear. Therefore, the present study was conducted to determine whether the expression levels of proinflammatory cytokines in peripheral nerves including the injured nerves and DRG are changed by EA that exerts analgesic effects.

## 2. Materials and Methods

### 2.1. Surgical Procedures

Adult male Spague-Dawley rats (220–250 g, *n* = 32) were used in this study. Animals were anesthetized with sodium pentobarbital (50 mg/kg, i.p.). A segment of the sciatic nerve was exposed between the mid-thigh and the popliteal fossa by skin incision and blunt dissection through the biceps femoris muscle. The three major divisions of the sciatic nerve (tibial, sural, and common peroneal nerves) were clearly separated based on individual perineuria. The tibial and sural nerves were tightly ligated and then transected, while the common peroneal nerve was left intact [15]. Complete hemostasis was confirmed and the wound was closed with muscle and skin sutures. There were four groups in this study: naïve (no surgery), sham operation (clearly separated sciatic nerve branches only), neuropathic surgery, and EA stimulation after neuropathic surgery. All animal experiments were performed in accordance with the policies and recommendations of the International Association for the Study of Pain and the National Institutes of Health guidelines for the handling and use of Laboratory animals and received approval from the Institutional Animal Care and Use Committee of Yonsei University Health System.

### 2.2. Behavioral Analysis

Behavioral tests to assess pain development were performed at postoperative days 1, 4, 7, and 14. To measure mechanical allodynia, rats were placed on a metal mesh floor under a custom-made transparent plastic dome (8 × 8 × 18 cm). Innocuous mechanical stimuli were applied to the sensitive area of each hind paw with a von Frey filament every 3-4 s (8 mN bending force, 10 repetitions). The frequency of foot withdrawal out of 10 trials with the von Frey filament application was expressed as a percentage (response rate (%) = number of foot withdrawals/number of trials × 100). To quantify cold sensitivity of the foot, brisk withdrawal in response to acetone applied to each paw every 5 min (5 repetitions) was documented. The frequency of foot withdrawal (expressed as a percentage) was used as a cold allodynia index.

### 2.3. EA Treatment

EA treatment was carried out after the behavioral test. The detailed methods for EA stimulation were described previously [2]. In short, rats were anesthetized with 2% enflurane in 95% O_2_/5% CO_2_. Stainless steel acupuncture needles (0.30 mm in diameter and 30 mm in length) were inserted percutaneously at a depth of 2-3 mm into the Zusanli (ST36) and Yinlingquan (SP9) acupuncture points. Electrical stimulation was produced by a stimulus isolation unit (A365, World Precision Instruments, Sarasota, FL, USA). Train pulses (0.6 mA, 1 Hz, 0.1 ms pulse width) were applied to the inserted needle for 10 min using the pulse master unit (A300, World Precision Instruments).

### 2.4. mRNA Quantitation Using Reverse Transcriptase

All rats were immediately sacrificed by decapitation after the final EA stimulation. The lumbar (L4-L5) DRG and the injured peripheral nerves were rapidly dissected and stored in 200 *μ*L lysis buffer (easy-BLUE reagent, iNtRON Biotechnology, Seoul, Republic of Korea). Total RNA (2 *μ*g) from each sample was reverse-transcribed into cDNA using SuperScript II Reverse Transcriptase (Invitrogen, Carlsbad, CA, USA). cDNA was amplified by polymerase chain reaction (PCR) in a 20 *μ*L reaction mixture containing 4 *μ*L dNTP mix (2.5 mM each), 1 *μ*L Oligo(dT) primer (500 ng/*μ*L), 4 *μ*L 5x first-strand buffer, and 2 *μ*L 0.1 M DTT. Reverse-transcript PCR (Mastercycler Gradient, Eppendorf, Hamburg, Germany) was performed for 37 cycles in a thermal cycler using PCR PreMix (Bioneer Inc., Alameda, CA, USA) under the following conditions: denaturation, 30 seconds at 95°C; annealing, 1 minute at 62°C; and extension, 2 minutes at 72°C. At the 37th cycle, extension was continued for an additional 5 minutes at 72°C. Reverse transcript PCR was performed for the amplification of IL-1*β*, IL-6, TNF-*α*, and *β*-actin (Table 1).

Amplified products were electrophoresed on 1.5% agarose gels, visualized with ethidium bromide, and photographed. To quantify band intensities, negative controls were scanned and then analyzed using the NIH image program. Amplification of endogenous *β*-actin was used to normalize IL-1*β*, IL-6, and TNF-*α* mRNA levels. 

### 2.5. Statistical Analysis

Values were expressed as the mean ± standard error of the mean (S.E.M) and compared using one-way or two-way ANOVA followed by Dunnett's *post hoc* pairwise comparison (SPSS Ver. 17.0, SPSS Inc., Chicago, IL, USA). A *P* value of less than 0.05 was considered significant.

## 3. Results

**Figure 1:**
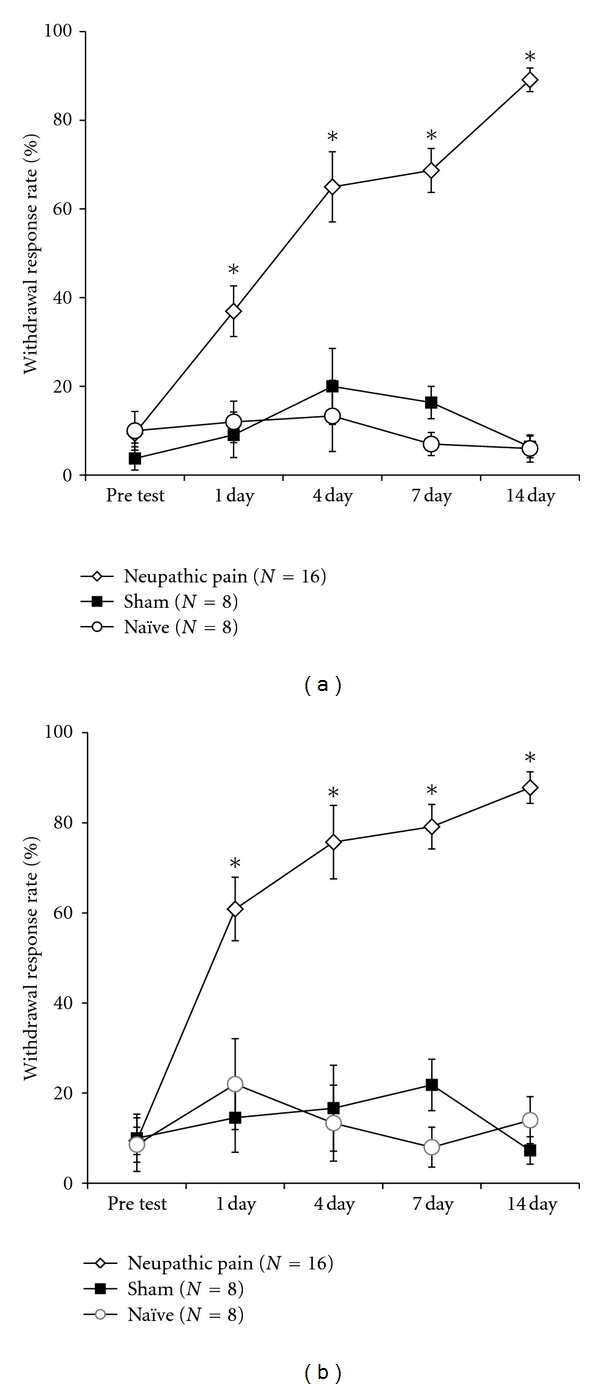
Development of mechanical (a) and cold allodynia (b) before EA stimulation. Naïve: the group without surgery; Sham: the sham-operated group; neuropathic: the neuropathic surgery group. Values are represented as mean ± SEM response rate as a percentage (number of foot withdrawals/number of trials × 100) (*P < 0.05 versus Naïve).

Behavioral signs of neuropathic pain were produced after nerve injury (Figure 1).  Figure 1(a) shows the development of mechanical allodynia observed before EA stimulation. The brisk withdrawal responses were induced when von Frey filament was applied to the receptive fields on nerve-injured hind paw. Two-way ANOVA showed significant main effects in between groups (*F*_2,7_ = 120.15, *P* < 0.01), and different time points (*F*_4,7_ = 12.82, *P* < 0.01), and significant interaction (*F*_8,7_ = 9.49, *P* < 0.01). This implies that behavioral performances in three groups are different depending on time points after surgery. Subsequent Dennett's* post hoc* multiple comparisons showed that neuropathic group displayed significantly higher withdrawal responses to von Frey filament at all time points after nerve injury (*P* < 0.05).  Figure 1(b) shows the development of cold allodynia observed before EA stimulation. The brisk withdrawal responses were induced when acetone was applied to the receptive fields on nerve-injured hind paw. Two-way ANOVA showed significant main effects in between groups (*F*_2,7_ = 54.23, *P* < 0.05), different time points (*F*_4,7_ = 7.59, *P* < 0.05), and significant interaction (*F*_8,7_ = 5.60, *P* < 0.05). This implies that behavioral performances in three groups are different depending on time points after surgery. Subsequent Dennett's* post hoc* multiple comparisons showed that neuropathic group displayed significantly higher withdrawal responses to acetone at all time points after nerve injury (*P* < 0.05). These behavioral results indicated that nerve-injured rats were more sensitive to stimulations and the sensitivity was continued until postoperative day 14.

**Figure 2:**
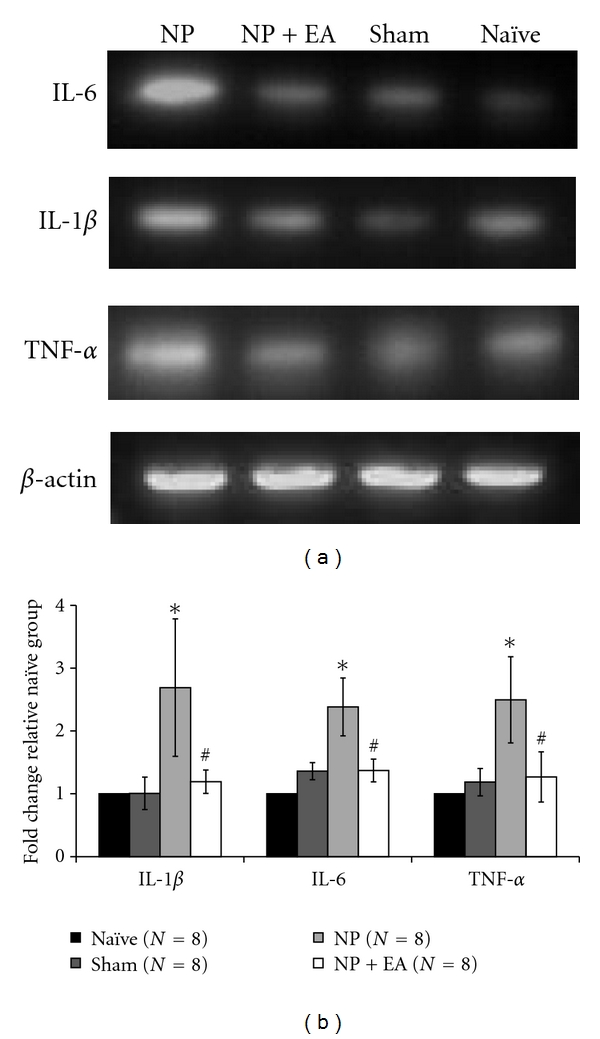
Effect of EA stimulation on cytokine mRNA expressions in injured nerves. (a) Representative photographs of cytokine expression in peripheral nerve tissue. (b) Expression levels of IL-1*β*, IL-6, and TNF-*α* in peripheral nerves of neuropathic rats were significantly higher than those in naïve rats. However, after EA stimulation, the cytokine expression levels were significantly decreased. NP: neuropathic group; NP+EA: neuropathic with electroacupuncture group; Sham: sham-operated group; Naïve: normal group  (*P < 0.05 compared to naïve, ^#^ *P* < 0.05 compared to neuropathic rats).

The expression of cytokines at the injured nerves ending following the development of pain was verified.  Figure 2(a) shows the expression levels of cytokine mRNAs (IL-1*β*, IL-6, and TNF-*α*) in four different groups. The neuropathic group (NP) showed higher cytokine mRNAs expression than Sham and Naïve groups. However, when EA was applied (EA+NP), the expression level was decreased.  Figure 2(b) shows the summarized results from 8 animals in each group. The neuropathic group only showed significant higher cytokine mRNAs expression levels for IL-1*β*, IL-6, and TNF-*α*. Furthermore, the EA application to neuropathic rats reduced the expression levels to the Sham and Naïve's level. 

**Figure 3:**
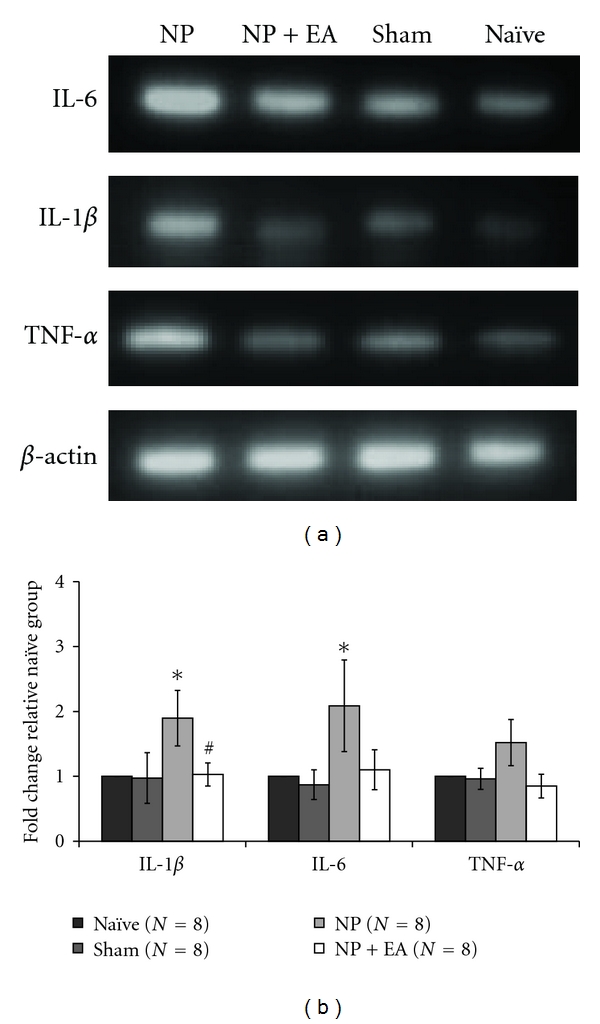
Effect of EA stimulation on cytokine mRNA expressions in the DRG. (a) Representative photographs of cytokine expressions in the DRG. (b) Expression levels of IL-1*β* and IL-6 in the DRG of neuropathic rats were significantly higher than those in naïve rats. However, the expression level of TNF-*α* was not significantly higher than in naïve rats. After EA stimulation, IL-1*β* cytokine expression level was significantly decreased. NP: neuropathic group; NP+EA: neuropathic with electroacupuncture group; Sham: sham-operated group; Naïve: normal group (*P < 0.05 compared to naïve,  ^#^ *P* < 0.05 compared to neuropathic rats).

The expressions of cytokines in the DRG were also observed. The DRG neurons in the neuropathic group showed higher cytokine mRNAs expression as the injured nerves did. The EA application reduced cytokine mRNA levels in the neuropathic rats (Figure 3(a)).  Figure 3(b) shows the summarized data in the DRG. The expression levels of IL-1*β* and IL-6 mRNAs were significantly higher in the neuropathic group than other groups. The expression of TNF-*α* tended to increase but the difference was not significant. EA application in neuropathic rats reduced the increased cytokine expressions. 

## 4. Discussion

As shown in the present study, neuropathic pain was developed following peripheral nerve injury. Our previous studies demonstrated that the behavioral signs of neuropathic pain can be alleviated by manual acupuncture or EA [2, 3]. According to Cha et al. [2], in particular pain-relieving effects of EA on mechanical allodynia lasted for 180 min (3 hrs) in neuropathic pain rats. However, the precise efficacy and mechanisms of analgesic effects of acupuncture stimulation for the treatment of neuropathic pain syndromes remain unclear.

Several lines of evidence indicate that acupuncture stimulation mediates the release of neurochemical factors at some sites in the central nervous system (CNS) [16]. EA produces an antihyperalgesic effect in a rat model of inflammatory pain by activating the spinal endorphin/endomorphin system (for *μ* receptors) and the enkephalin system (for *δ* receptors) [17]. Serotoninergic and noradrenergic systems of pain inhibition may also mediate the analgesic effects of acupuncture [10, 13, 18–20]. Cha et al. [2] also reported that EA inhibits the expression level of nitric oxide synthase in the spinal cord of neuropathic rats. These studies suggest that EA stimulation may produce neurochemical changes in the nervous system.

Cytokines belong to glycoproteins which have low molecular weight. These are secreted by immune cells like T-cells, macrophages, and neutrophils. In the nervous system, Schwann cells and glial cells can synthesize and release cytokines [21, 22]. mRNA for proinflammatory cytokines including TNF-*α*, IL-1*β*, and IL-6 has been shown to increase in the DRG and spinal cord [14].

Recently, the relationship between cytokine expressions and neuropathic pain was reported. Evidence shows that cytokine signaling is related to pain development. For example, the time-dependent spinal cytokine expression levels showed a rapid and transient upregulation of TNF-*α* mRNA, a delayed upregulation of IL-1*β* mRNA, and a rapid and more sustained upregulation of IL-6 mRNA in the spinal nerve ligation model of neuropathic pain [23, 24]. Furthermore, it has been known that IL-1*β* and TNF-*α* are important for the initiation of persistent neuropathic pain, and IL-6 functions to maintain it [14]. Our results also showed significant increase in mRNA levels of proinflammatory cytokines in the injured nerves and DRG following the development of neuropathic pain.

However, the expression levels of TNF-*α* tended to increase but were not significantly high compared to the controls in DRGs while the expression levels significantly increased in the injured nerve. The TNF-*α* signaling pathway is activated early in abnormal pathological pain and plays a pivotal role in initiation of the proinflammatory cytokine cascades including IL-1*β* and IL-6 in the nervous system including the DRG [25, 26]. It is not easy to explain the reason why there is no significant increase of TNF-*α* mRNA in the DRG. According to Lee et al. [14], TNF-*α* mRNA levels were immediately elevated in the DRG at 1 day after nerve injury and then gradually reduced. The expression levels of TNF-*α* mRNA were not different from the control group by 7 days after injury. Because the cytokine levels were investigated after behavioral test for 14 days, this may explain, at least in part, the lack of significant increase in TNF-*α* mRNA levels in nerve injured rats.

To date, however, the effects of acupuncture on proinflammatory cytokine expressions were not studied systematically yet. In the present study, we demonstrated that the expression levels of proinflammatory cytokines were dramatically decreased after EA stimulation in both the injured peripheral nerves and DRG of neuropathic rats. There were some studies which showed the effect of EA on pain attenuation and inhibition of cytokines expression in the central nervous system using an animal model of cancer pain [27] and on reduction of inflammation-induced cytokine expression by acupuncture using an animal model of carrageenan-induced inflammatory pain [28]. Similar to cancer pain and inflammatory pain, we demonstrated that EA inhibits the expressions of proinflammatory cytokines.

In summary, our results showed that the expression of cytokines in the DRG and injured peripheral nerves dramatically increased in neuropathic rats and significantly decreased after EA stimulation. This suggests that the increased levels of cytokines may be related to persistent pain which can be modulated by acupuncture stimulation including EA. However, the detailed mechanisms involved in cytokine expression related to acupuncture analgesia remain to be determined. A better understanding of the neurobiological mechanisms of EA stimulation-mediated analgesia may help to improve its effectiveness.

## 5. Conclusions

We generated neuropathic model and confirmed its sensitivity to pain behaviorally. The pain signaling significantly increased the cytokine levels in the injured nerves and DRG in neuropathic group than Sham and Naïve groups. But these increased cytokine levels were reduced by EA application after pain generation. The results indicate that EA stimulation can reduce the inflammatory cytokine expressions through pain signaling and modulation pathways. These suggest that EA stimulation is effective in the modulation of the inflammatory cytokine expression and it maybe an effective analgesic treatment on neuropathic pain symptoms.

## Figures and Tables

**Table 1:** Primers used in reverse transcript PCR.

Gene name	Primer sequence
IL-1*β*	F: 5′-GGAAGGCAGTGTCACTCATTGTG-3′ R: 5′-GGTCCTCATCCTGGAAGCTCC-3′
IL-6	F: 5′-GGGACTGATGTTGTTGACAGCC-3′ R: 5′-CATATGTAATTAAGCCTCCGACTT-3′
TNF-*α*	F: 5′-CCCCGACTATGTGCTCCTCAC-3′ R: 5′-AGGGCTCTTGATGGCGGA-3′
*β*-actin	F: 5′-TGGAATCCTGTGGCATCCATGAAAC-3′ R: 5′-TAAAACGCAGCTCAGTAACAGTCCG-3′

(F: Forward primer; R: Reverse primer).
